# Importance of Polη for Damage-Induced Cohesion Reveals Differential Regulation of Cohesion Establishment at the Break Site and Genome-Wide

**DOI:** 10.1371/journal.pgen.1003158

**Published:** 2013-01-10

**Authors:** Elin Enervald, Emma Lindgren, Yuki Katou, Katsuhiko Shirahige, Lena Ström

**Affiliations:** 1 Department of Cell and Molecular Biology, Karolinska Institute, Stockholm, Sweden; 2 Research Center for Epigenetic Disease, Institute of Molecular and Cellular Biosciences, The University of Tokyo, Tokyo, Japan; University of California Berkeley, United States of America

## Abstract

Genome integrity depends on correct chromosome segregation, which in turn relies on cohesion between sister chromatids from S phase until anaphase. S phase cohesion, together with DNA double-strand break (DSB) recruitment of cohesin and formation of damage-induced (DI) cohesion, has previously been shown to be required also for efficient postreplicative DSB repair. The budding yeast acetyltransferase Eco1 (Ctf7) is a common essential factor for S phase and DI-cohesion. The fission yeast Eco1 ortholog, Eso1, is expressed as a fusion protein with the translesion synthesis (TLS) polymerase Polη. The involvement of Eso1 in S phase cohesion was attributed to the Eco1 homologous part of the protein and bypass of UV-induced DNA lesions to the Polη part. Here we describe an additional novel function for budding yeast Polη, i.e. formation of postreplicative DI genome-wide cohesion. This is a unique Polη function not shared with other TLS polymerases. However, Polη deficient cells are DSB repair competent, as Polη is not required for cohesion locally at the DSB. This reveals differential regulation of DSB–proximal cohesion and DI genome-wide cohesion, and challenges the importance of the latter for DSB repair. Intriguingly, we found that specific inactivation of DI genome-wide cohesion increases chromosomal mis-segregation at the entrance of the next cell cycle, suggesting that S phase cohesion is not sufficient for correct chromosome segregation in the presence of DNA damage.

## Author Summary

Correct chromosome segregation requires that sister chromatids are held together by the protein complex cohesin, from S phase until anaphase. This S phase established cohesion is, together with DSB recruitment of cohesin and formation of damage-induced (DI) cohesion, also important for repair of DSBs. Eco1 is a common essential factor for S phase and DI-cohesion. The fission yeast Eco1 ortholog, Eso1, is important both for S phase cohesion and for bypass of UV-induced lesions, and is expressed as a fusion protein with Polη. The cohesion function has been attributed solely to Eso1 and the lesion bypass function to the Polη part of the protein. As we found the interaction between the two proteins intriguing, we decided to look for a functional connection also in budding yeast. Indeed, despite being dispensable for S phase cohesion, budding yeast Polη is required for formation of DI genome-wide cohesion. However, Polη-deficient cells are DSB repair competent, revealing differential regulation of DI-cohesion at the break and genome-wide. This finding challenges the importance of DI genome-wide cohesion for DSB repair, and based on our findings we suggest that S phase cohesion is not sufficient for correct chromosome segregation in the presence of DNA damage.

## Introduction

Correct chromosome segregation is fundamental for genome integrity, and facilitated by the cohesin complex, that tethers sister chromatids from S phase until anaphase, a function known as cohesion [1], [2], [3]. Cohesin consists of four subunits: Smc1, Smc3, Scc1 (also called Mcd1), and Scc3 and associates with DNA prior to replication [4], [5], [6], [7]. In all organisms analyzed to date, loading of cohesin onto chromosomes requires a complex formed by the Scc2 and Scc4 proteins [8], [9]. However, loading alone is not sufficient for actual sister chromatid cohesion to commence. Cohesion is established during S phase in an incompletely understood process that is closely connected with replication and depends on acetylation of Smc3 by the highly conserved acetyltransferase Eco1 (also called Ctf7) [10], [11], [12]. Several proteins have been shown to be important for cohesion establishment, including Ctf18, a subunit of an alternative replication factor C (RFC) complex and the proliferating cell nuclear antigen (PCNA), [13], [14], [15], [16]. Once established, cohesion is maintained until anaphase, when it is dissolved through cleavage of Scc1 by the enzyme separase (for a review see [3]).

DNA double strand breaks (DSBs) can arise during normal cellular processes such as replication stress and replication fork collapse, as well as programmed genomic rearrangements including yeast mating-type switching, immunoglobulin class-switch recombination and DSB induction during meiotic prophase [17], [18]. Evidently, DNA damage can also be a consequence of exposure to DSB inducing agents such as ionizing radiation and various chemicals [17]. Regardless, correct repair of damaged DNA is vital for genome integrity. Cohesion formed during S phase is required for postreplicative repair of DSBs via homologous recombination (HR) [19], [20]. In addition to S phase cohesion, recruitment of cohesin to the region around the DSB and formation of cohesion genome-wide, a phenomenon called damage induced (DI)-cohesion, has been shown to be important for DSB repair [21],[22],[23]. The establishment of DI-cohesion requires a number of proteins, such as the cohesion regulatory factors Scc2 and Eco1, the Smc5/6 complex, the DNA-damage sensing protein Mre11, the checkpoint kinases Mec1/Tel1, phosphorylated H2A, and activation of the Mec1 target Chk1 [24]. In addition, DI-cohesion has been proposed to depend specifically on acetylation of Scc1 via Eco1 [25].

Of the factors required for both S phase and DI-cohesion, Eco1 has been shown to be a limiting component [23]. Interestingly, in the fission yeast *Schizosaccharomyces (S.) pombe*, the Eco1 ortholog Eso1 is expressed as a fusion protein with the translesion synthesis (TLS) polymerase Polη [26]. Eso1 is required for the establishment of cohesion but deletion mutants of Eso1 that lack the Polη-containing N-terminal part are effectively S phase cohesion proficient [26]. An additional link between Polη and cohesin is the S phase cohesion establishment factor Ctf18 that has been shown to exclusively activate Polη in *Saccharomyces (S.) cerevisiae* [16], [27].

TLS polymerases are found in all domains of life [28], [29] and are best known for their ability to bypass DNA damage that blocks the replication fork progression [30]. Since TLS polymerases have active sites with more open structures than the replicative DNA polymerases, they can bind to and replicate past DNA with aberrant structures [31]. *S. cerevisiae* has three TLS polymerases Rev1, Polζ (Rev3/7) and Polη [28], [29]. The gene encoding Polη is in *S. cerevisiae* called *RAD30*. This gene nomenclature was proposed based on the original finding that a *RAD30* deletion causes UV-sensitivity [32]. Both Polη and Rad30 are seen as names for the protein in *S. cerevisiae*, while the human ortholog is called Polη. Here we are therefore using Polη as a common name for the protein while *RAD30* is used when we are discussing the yeast gene. The function of Polη has been best characterized in the process that inserts appropriate nucleotides opposite UV induced cis-syn cyclobutane pyrimidine dimers (CPDs), a type of DNA damage that typically blocks the progression of the replication fork since the highly stringent replicative DNA polymerases are unable to bypass it [33], [34], [35], [36]. Patients with the Xeroderma pigmentosum variant disease (XP-V), caused by loss of Polη function, display a higher rate of UV-induced mutations and a greatly increased incidence of skin cancer [37], [38].

Here, we investigated the functional relationship between Polη and Eco1 in *S. cerevisiae*. We found that in the absence of Polη, the establishment of DI-cohesion is abolished. This deficiency could be counteracted by overexpression of *ECO1*, as well as by an acetyl-mimic version of one of the Eco1 acetylation targets, *SCC1 (pGAL-scc1-K84Q, K210Q)*, indeed suggesting that Polη is important for the function of Eco1. Despite the importance for DI-cohesion, *RAD30* deleted cells are fully capable of postreplicative DSB repair during G2. This could be explained by the findings that Polη is essential explicitly for DI genome-wide cohesion and not for loading of cohesin to the break, or for formation of DI-cohesion close to the actual DSB. In summary, this not only reveals that cohesion in response to DNA damage is regulated differently close to the break and genome-wide, but it also challenges the functional importance of the genome-wide form. Our study indicates that lack of DI genome-wide cohesion causes a predisposition to chromosomal mis-segregation at the entrance to the next cell cycle, which after exposure to repeated DSB inductions seems to have negative consequences for survival.

## Results

### Polη is required for formation of DI-cohesion in response to γ-irradiation as well as to induction of a single DSB

**Figure 1:**
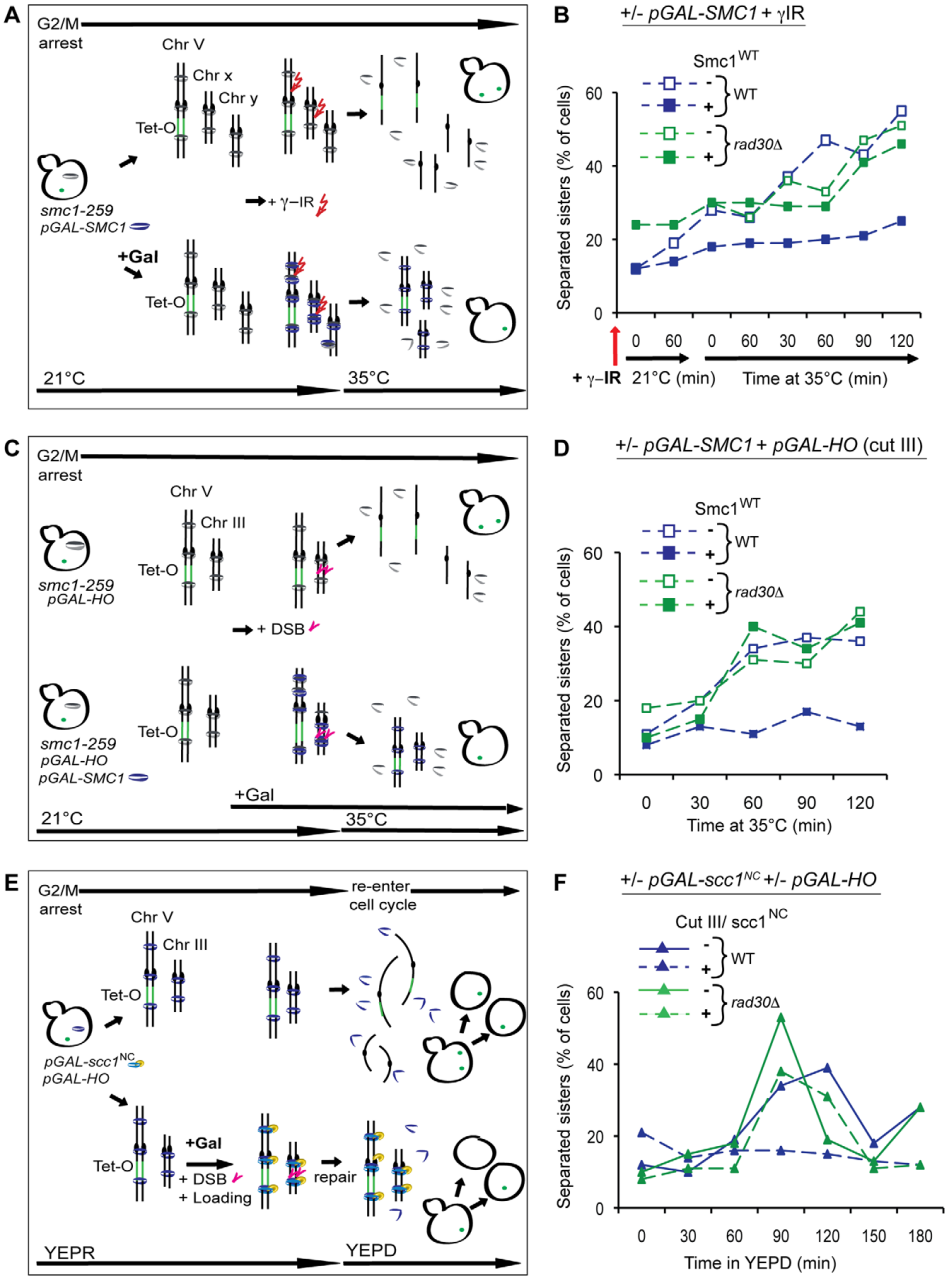
Polη is required for formation of cohesion in response to DSB during G2. (A) The experimental system used for detection of DI genome-wide cohesion activated by γ-IR. Cells harboring the temperature sensitive (ts) *smc1-259* allele together with *pGAL-SMC1* (*smc1^ts^*/Smc1^WT^ system) were grown in YEP media supplemented with 2% raffinose (YEPR) at 21°C, arrested in G2/M by addition of benomyl and kept at G2/M throughout the experiment. Expression of *pGAL-SMC1* was induced in one half of the culture by addition of galactose (2%). DNA damage was induced by to γ-IR (350 Gy), and loading of cohesin and establishment of DI-cohesion allowed during 60 min at permissive temperature. The temperature was raised to 35°C whereby cohesion created during S phase, or after DNA damage by *smc1^ts^* was degraded. γ-IR is denoted by a red arrow. (B) Detection of DI-cohesion after γ-IR using the s*mc1^ts^*/Smc1^WT^ system, as in A, in WT or *rad30Δ* strains. Samples for analyzis of sister separation at the *URA3* locus on Chr. V by the TetR-GFP/Tet-O system were collected, and separation scored in ≥200 cells/time point. A red arrow denotes time point for γ-IR. Blue lines represent WT and green lines *rad30Δ* cells. Dashed lines indicate damage induction. (C) System for detection of DI genome-wide cohesion induced by a single DSB, in cells harboring the *smc1^ts^* allele and the *pGAL-HO* that creates a DSB at the *MAT* locus on Chr. III. These cells are compared with cells in addition containing *pGAL-SMC1*. Cells were arrested in G2/M (as in A), when galactose was added to induce the expression, if present, of *pGAL-SMC1* and *pGAL-HO*. After 90 min, the temperature was raised to 35°C to destroy cohesion created during S phase or after DNA damage by *smc1^ts^*. Induction of a DSB on Chr. III is indicated by magenta arrowheads. (D) DI-cohesion was determined after induction of a DSB induced at the *MAT* locus on Chr. III by expression of *pGAL-HO* as described in C, otherwise as in B. (E) Assay for detection of DI genome-wide cohesion formed by a non-cleavable version of Scc1 (Scc1^NC^), expressed from the *GAL* promoter (*pGAL-scc1^NC^*). Cells grown in YEPR at 25°C were arrested in G2/M as in A. Galactose addition to one half of the culture induced expression of *pGAL-HO* and *pGAL-scc1^NC^*. Cohesin loading and cohesion formation was allowed during 90 min, when the cells were released into YEPD to allow repair and re-entry into the cell cycle. Provided the DSB was repaired, cells re-entered the cell cycle and went through mitosis. DI-cohesion formed via Scc1^NC^ prevented sister separation regardless. Eventually also these cells entered G1 but then with mis-segregated chromosomes. Induction of a DSB on Chr. III is indicated by magenta arrowheads. (F) Formation of DI-cohesion was examined using the Scc1^NC^ system as in 1E otherwise as in B and D.

To investigate whether Polη is important for formation of DI-cohesion, the *S. cerevisiae RAD30* gene that encodes Polη was deleted (*rad30Δ*) in strains harboring systems that allow distinction between S and G2 phase established cohesion, described in Figure 1A, 1C and 1E and below. All the assays used for detection of DI-cohesion are based on the Tet-repressor-GFP/Tet-operators (TetR-GFP/Tet-O) system for sister chromatid separation. This system utilizes the insertion of an array of Tet-operators at the *URA3* locus, 38 kb from the centromere of Chromosome (Chr.) V, to which the endogenously expressed GFP-tagged Tet-repressor will bind. This results in one GFP focus in cells where the sisters are cohered and two foci where they are separated [21],[22],[23]. Initially we tested whether Polη was required for the formation of DI-cohesion in response to γ-irradiation (γ-IR). Log phase yeast cells harboring a temperature sensitive (ts) *SMC1* allele (*smc1-259 or smc1^ts^*) were arrested in G2/M by addition of benomyl. Expression of *pGAL*-*SMC1* (Smc1^WT^) was initiated at permissive temperature in one half the cultures and DNA damage was induced by γ-IR, the cells were then allowed one hour to recruit cohesin, containing either *smc1^ts^* or Smc1^WT^, to DNA and to establish DI-cohesion. Thereafter, incubation at restrictive temperature caused degradation of the cohesion formed by *smc1^ts^* (Figure 1A). As seen in Figure 1B, and as shown previously [21], DI-cohesion was formed in response to γ-IR only when Smc1^WT^ was expressed. However, DI-cohesion was strongly disabled in the absence of Polη.

DI-cohesion has also been shown to arise genome-wide in response to a single DSB [22], [23]. To examine whether lack of Polη would also prevent establishment of cohesion under these conditions, we used the *smc1^ts^*/Smc1^WT^ system (Figure 1A) in combination with expression of the galactose-inducible, site specific, HO-endonuclease (*pGAL-HO*), which induced a single DSB at the *MAT* locus on Chr. III (Figure 1C). Because the Tet operators are located on Chr. V, any DSB-dependent cohesion that is observed must be genome-wide. Indeed, in the absence of Polη establishment of DI-cohesion was impaired also in response to one single DSB (Figure 1D). To exclude that the effect seen on DI-cohesion was due to the combination of *rad30Δ* and *smc1-259*, we took advantage of a noncleavable version of the Scc1 subunit (Scc1^NC^) of the cohesin complex which cannot be cleaved off the chromosomes by separase (Figure 1E) [39]. In this system for detection of DI-cohesion, cells are arrested in G2/M, and expression of *pGAL-HO* and *pGAL*-*scc1^NC^* are initiated by addition of galactose in half of the cultures. After 90 minutes, when cohesin has been loaded and cohesion formed in response to the DSB, the cells are released from the G2 arrest, by transfer into YEPD media, whereby also the break formation is stopped. The single DSB can then be repaired and the cells re-enter the cell cycle, go through anaphase and separate their sisters, unless DI-cohesion has been established using Scc1^NC^. Again, in wild-type cells DI-cohesion is established after DSB induction and expression of Scc1^NC^, while in *rad30Δ* cells it is not. We could also conclude that lack of DI-cohesion in the absence of Polη was not due to deficient S phase cohesion, caused by the combination of the *smc1^ts^* allele and the *rad30Δ* (Figure 1F). Further evidence for an unperturbed S phase progression, was given by FACS analyses and determination of cell population doublings in comparison with WT cells (data not shown and Figure S1). From these results we propose that functional Polη is indeed required for DI-cohesion.

### Despite its importance for DI cohesion, Polη is not required for DSB repair in postreplicative cells

**Figure 2:**
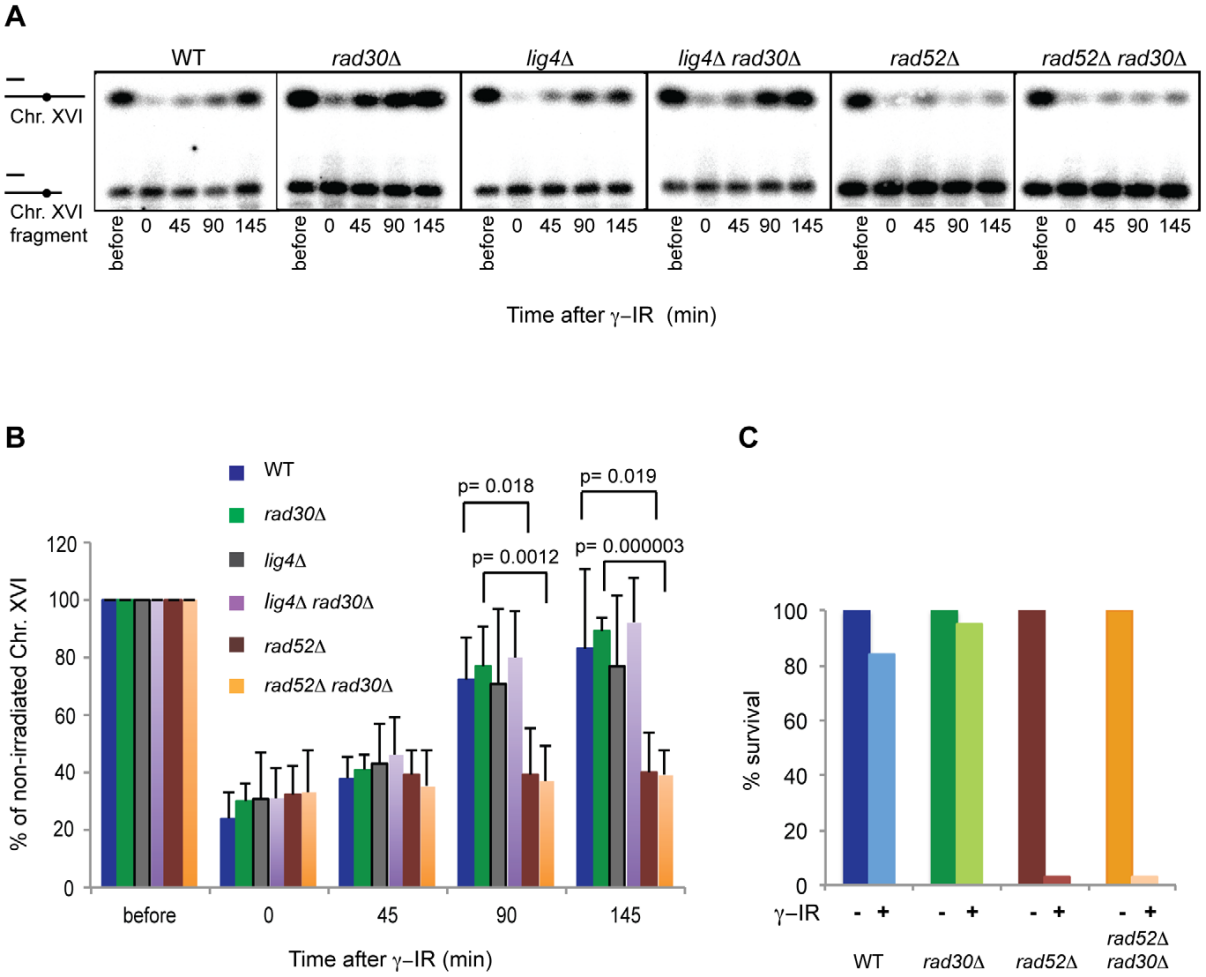
Polη is not required for homologous recombination-mediated postreplicative DSB repair. (A) Representative examples of pulsed field gels run for analysis of DSB repair efficiency. G2/M arrested cells with the indicated genes deleted were isolated before and at indicated time points following 150 Gy γ-IR. Cells were lysed in agarose plugs and genomic DNA was resolved by pulsed field gel electrophoresis. Control cells containing two fragments of Chr. XVI (0–685 and 685–948 kb from left telomere) were added as loading control [61]. After Southern blotting, membranes were hybridized with a Chr. XVI probe, containing Chr. XVI left arm specific sequences. Approximate region of association with Chr. XVI is shown as a black bar above indication for Chr. XVI. The fraction of intact Chr. XVI in relation to control Chr. XVI was quantified using a phosphoimager. (B) Averages from quantification of pulse field gels as in (A) from ≥3 exp for each strain. The results are presented as the ratio of the intact Chr. XVI remaining at the specified time compared with unirradiated Chr. XVI measured before γ-IR, related to the fragmented Chr. XVI in each lane. Error bars represent standard deviation (SD) based on a minimum of three individual experiments and significant differences are indicated with *p*-values (students *t-test*). (C) G2/M arrested cells with indicated genotypes were exposed to 150 Gy γ-IR after which 300 cells were plated on YEPD plates. After 2 days colonies were counted for estimation of survival. The results are shown as the fractions of colonies surviving after γ-IR compared with non-irradiated controls of the same genotype. One representative experiment (from two performed) is shown.

Based on previous experiments where the function of Eco1 was inactivated, DI-cohesion was concluded to be important for efficient repair of DSBs in G2 [22], [23]. After observing that Polη was required for establishment of DI-cohesion, we analogously wanted to investigate whether it was also important for postreplicative DSB repair. G2/M arrested WT and *rad30Δ* cells were exposed to γ-IR and thereafter allowed time for repair of induced damage. The dosage applied to the cells caused an approximate 70% reduction of the signal of intact Chr. XVI immediately after γ-IR (Figure 2A–2B), as analyzed by pulse field gel electrophoresis (PFGE) and Southern blotting with a radioactive probe hybridizing to the left arm of Chr. XVI [19]. Over time this signal was restored to the same extent in WT and *rad30Δ* cells indicating that Polη, despite its importance for formation of DI-cohesion, was of no significance for repair of the damage that activates the genome-wide cohesion (Figure 2A–2B). We then wanted to exclude that the repair was performed via Non-Homologous End Joining (NHEJ), as an alternative to HR in the absence of DI-cohesion. The *LIG4* gene encoding ligase 4, essential for NHEJ [40], was deleted (*lig4Δ*), either alone or in combination with *rad30Δ*. In both cases DSB repair was as efficient as in WT cells indicating that postreplicative DSB repair can occur via HR also in the absence of Polη (Figure 2A–2B). As a control we also analyzed the level of repair in cells where the *RAD52* gene, absolutely required for HR [41], was deleted *(rad52Δ)* either alone or in combination with *rad30Δ*, which caused a complete absence of repair as expected (Figure 2A–2B). To exclude that the HR mediated repair performed in the absence of Polη was the result of a defective mode of repair incompatible with life, we analyzed survival after γ-IR by the ability to form colonies and found that >80% of both WT and *rad30Δ* cells survived. This was in strong contrast to the less than 1% of cells that survived in the *rad52Δ* population after exposure to the same radiation dosage (Figure 2C). Thus, despite its importance for establishment of DI-cohesion, Polη was dispensable for repair of DSBs induced by γ-IR in the G2 phase.

### Genome-wide and DSB proximal DI-cohesion are regulated differently

**Figure 3:**
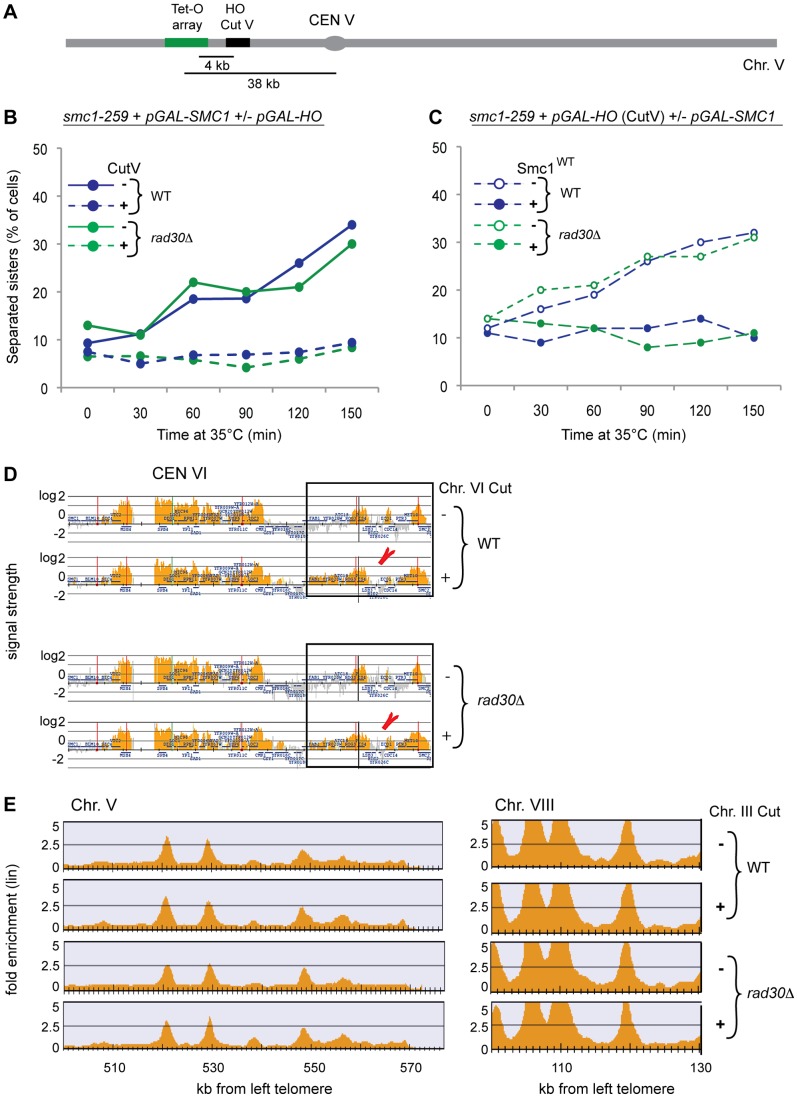
Polη is required for DI genome-wide cohesion but not for cohesin loading or establishment of DSB–proximal cohesion. (A) Schematic illustration of Chr. V with the Tet-O array and the inserted HO cut-site (not to scale). (B) Formation of Chr. V DSB-proximal cohesion was examined in WT and *rad30Δ* cells using the *smc1^ts^*/Smc1^WT^ system (Figure 1C). Smc1^WT^ was expressed in all groups, and a DSB induced 4 kb from the Tet-O array on Chr. V, in half of the cultures, by expression of *pGAL-HO*. Blue lines represent WT and green lines *rad30Δ* cells. Dashed lines indicate damage induction. (C) The Chr. V DSB was induced in all groups and Smc1^WT^ expressed in indicated sample series, otherwise as in B. (D) Scc1-FLAG binding determined by ChIP on chip after DSB induction for 90 min on Chr. VI (206 kb from the left telomere, indicated by red arrow heads) in G2/M arrested cells of indicated genotype. Orange peaks display significant chromosomal binding sites where the x-axis show chromosomal positions and the y-axis show log_2_ of signal strength. (E) Chromosomal association of G2-expressed Scc1-HA analyzed by ChIP-sequencing in WT and *rad30Δ* cells in the presence and absence of DSB induction at the *MAT* locus on Chr. III. Shown are, to the left Chr. V 500–570 kbp and to the right Chr. VIII 100–130 kb, from the left telomeres respectively. Orange peaks display significant chromosomal binding sites, the x-axis show chromosomal positions and the y-axis show linear fold enrichment.

The assays used for detecting DI-cohesion are based on induction of either a single DSB on Chr. III by expression of *pGAL-HO*, or multiple DSBs randomly distributed throughout the genome by γ-IR, but causing approximately one break/Chr. V. Given that Chr. V is 540 kbp, a break would only rarely be formed in the direct vicinity of the TetR-GFP/Tet-O system at the *URA3* locus, which is used for determining cohesion (Figure 1A, 1C and Figure 3A). Thus, one possible explanation for why absence of Polη did not lead to DNA repair deficiency, despite causing defects in formation of DI-cohesion, could be that DI-cohesion genome-wide and close to the actual break are regulated differently. If this is true, cohesion that could be used for HR-based repair would still be established locally around each DSB in the absence of Polη. To test this, we inserted the recognition sequence for the HO enzyme 4 kb from the Tet-O array, and simultaneously deleted the endogenous recognition sequence at the MAT locus on Chr. III, in cells harboring the *smc1^ts^*/Smc1^WT^ system for detecting DI-cohesion (Figure 3A). In the presence of Smc1^WT^, cohesion was now established both in WT and *rad30Δ* cells in response to, and close to a specific DSB (Figure 3B). DI-cohesion was formed only in the presence of Smc1^WT^ confirming that this was due to presence of Smc1^WT^, rather than to sisters being kept together due to lack of DSB repair (Figure 3C). Since DI-cohesion around the break and genome-wide seems to be regulated differently, from now on we will call them DSB-proximal cohesion and DI genome-wide cohesion respectively.

We next asked whether also cohesin loading genome-wide and close to the break were regulated differently. In line with the lack of importance for establishment of DSB-proximal cohesion, recruitment of cohesin to the break in the absence of Polη was not affected (Figure 3D). This was shown using chromatin immunoprecipitation combined with microarrays (ChIP on chip) on FLAG-tagged Scc1, as described previously [21]. Furthermore, in the same type of experiments FLAG-tagged Polη could not be detected at or close to the DSB between 10 and 120 minutes after break induction (data not shown). This was not because Polη could not be detected by ChIP on chip since Polη binding was detected at some replication origin sites during S phase arrest (Figure S2). We then examined possible differences in loading of cohesin to regions distant from a DSB, in WT and *rad30Δ* cells. To be able to distinguish between S phase and G2 phase loaded cohesin we performed ChIP-sequencing experiments on HA-tagged Scc1, expressed from the *GAL* promoter, specifically in G2. The genome-wide binding pattern of G2 loaded Scc1 was virtually identical in WT and *rad30Δ* cells and overlapped with the known binding pattern of cohesin, both in the presence and absence of DSB induction (Figure 3E, and Figure S3A) [42]. Recent data suggested that DNA damage causes an approximately 30% enhancement of cohesin binding genome-wide in human cells [43]. To exclude quantitative differences in cohesin binding between WT and *rad30Δ* cells, G2 loaded Scc1 binding was determined at a number of known cohesin association sites (CARs) in two selected regions of the genome, as well as at one non-binding site, using ChIP in combination with real time quantitative PCR (qChIP). At the previously identified cohesin non-binding site, significantly less cohesin was detected than at CARs. However, we could not detect any significant changes in cohesin binding at the known CARs on Chr. V and VIII that we analyzed, neither in WT nor in *rad30Δ* cells before or after induction of a single DSB on Chr. III (Figure S3B). Thus, despite that DSB-proximal and DI genome-wide cohesion are differently regulated, the basis for this disparity cannot be attributed to differences in loading of cohesin.

### Polη's function in DI-cohesion is unique and not common to all translesion synthesis polymerases

**Figure 4:**
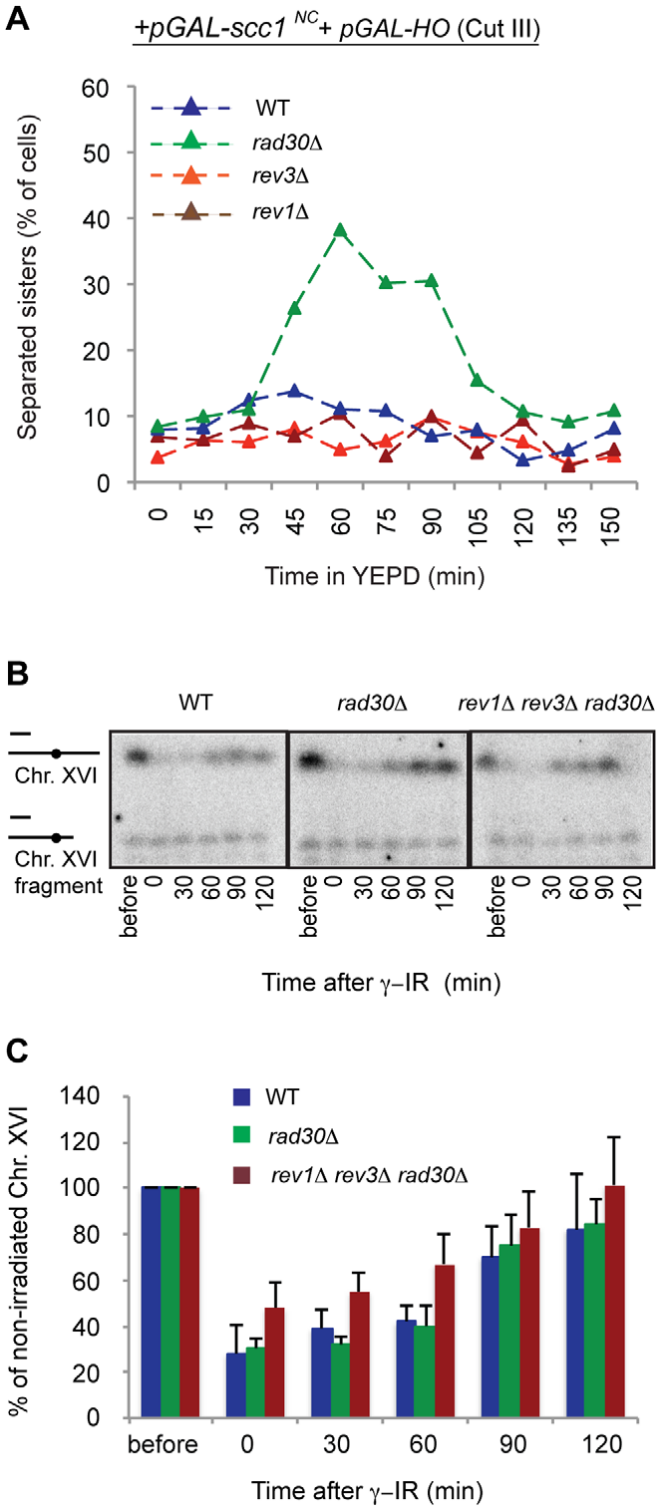
Establishment of DI-cohesion is a function unique for Polη among the TLS polymerases. (A) Using the Scc1^NC^ system (Figure 1E), establishment of DI-cohesion was compared between WT, *rev1Δ*, *rev3Δ* or *rad30Δ* deletion mutants. One DSB at the *MAT* locus on Chr. III was induced by *pGAL-HO*. Percentage of separated sister chromatids were scored in ≥200 cells per time point by the TetR-GFP/Tet-O-system (see Figure 1). (B) Determination of DSB repair during G2 in a triple TLS polymerase deletion strain by PFGE (See Figure 2A for details). (C) Averaged quantifications of pulsed field gels run on the indicated strains (See Figure 2B for details).

Given that Polη is required for DI genome-wide cohesion, we decided to test whether this is a common function for TLS-polymerases. Strains were created with deletions of the *REV1* or the *REV3* gene (which encodes the catalytic subunit of Polζ) and DI-cohesion experiments were performed using the Scc1^NC^ system and induction of one break by HO (Figure 1E). As seen in Figure 4A, neither the Rev1 (*rev1Δ*) nor the Polζ (*rev3Δ*) polymerase was of any importance for DI genome-wide cohesion in response to a single DSB. In addition, none of the TLS polymerases were required to establish cohesion during S phase, as seen by lack of sister chromatid separation on arrival in G2/M. The possibility that Rev1 and/or Polζ could replace Polη at the break for DSB-proximal cohesion was excluded by the fact that even in a triple-deletion strain (*rev1Δ rev3Δ rad30Δ*) efficient DSB repair was observed (Figure 4B–4C). Thus the function for Polη in DI genome-wide cohesion is not shared between the TLS polymerases.

### The polymerase activity of Polη is dispensable for DI-cohesion

**Figure 5:**
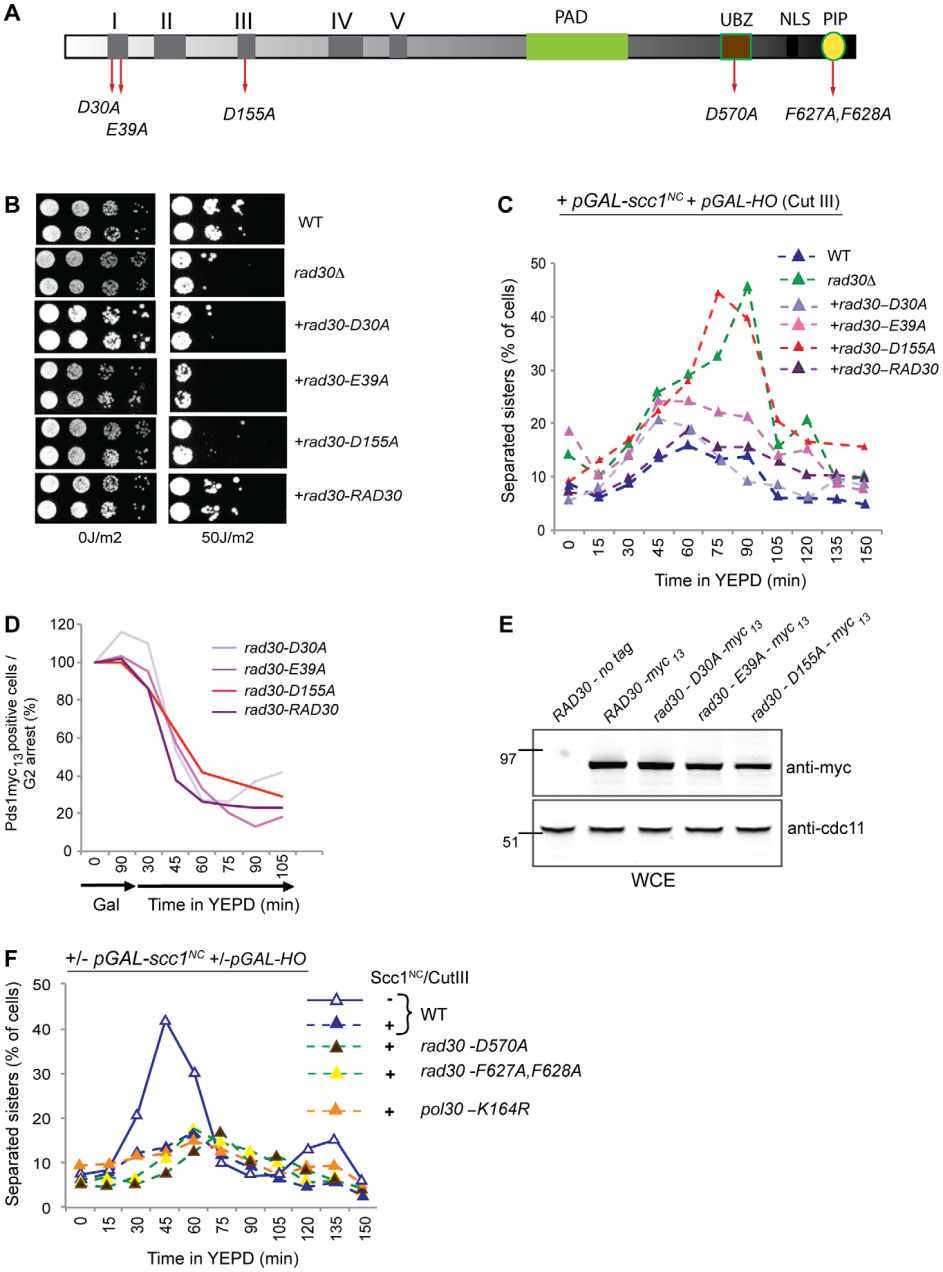
The DI-cohesion function of Polη is not mediated via its polymerase activity or through PCNA-interaction. (A) Polη with relative positions of the five conserved polymerase motifs (I–V), the polymerase associated domain (PAD), the ubiquitin-binding motif (UBZ), the nuclear localization signal (NLS) and the PCNA-binding box (PIP). Mutated residues analyzed in this study are indicated with arrows (not to scale). (B) UV-sensitivity was analyzed in *rad30Δ* cells carrying the *RAD30 or rad30*-*D30A*, -*E39A* or -*D155A* genes on a low copy-number *CEN LEU2* plasmid. Exponentially growing cells were plated on YEPD plates in 10-fold dilutions, left untreated or UV irradiated (50 J/m^2^) as indicated, and then incubated 3 days at 25°C. (C) DI-cohesion measured with the Scc1^NC^ system (Figure 1E) in response to one DSB on Chr. III in WT and *rad30Δ* cells in comparison with *rad30Δ* cells carrying the *RAD30*, *rad30*-*D30A*, -*E39A* or -*D155A* plasmids. Sister chromatid separation was scored with the TetR-GFP/Tet-O-system. (D) *rad30Δ* strains harboring polymerase mutant versions of *rad30* exit the G2/M arrest simultaneously. Percentages of Pds1-myc_13_ positive cells were determined by *in situ* staining with an anti-myc antibody. The percentage of positive cells at each time point is related to the percentage at complete G2 arrest for each strain, which is set to 100%. (E) Polη-myc_13_ levels were analyzed by Western blotting. Whole cell extracts were isolated from WT cells (*RAD30-no tag*), cells harboring *RAD30-myc_13_* or cells with differently mutated *rad30-myc_13_* tagged alleles as indicated. Cdc11 was used as loading control. One representative experiment (from four performed) is shown. (F) The requirement for Polη interaction with PCNA in DI-cohesion was tested in *pol30*-*K164R*, *rad30*-*D570A* and *rad30*-*F627A*, *F628A* mutants using the Scc1^NC^ system (Figure 1E).

We next sought to understand the mechanism by which Polη supports formation of DI genome-wide cohesion. Since Polη is defined as a polymerase we naturally started by testing whether the TLS polymerase activity was required [28], [29]. Plasmids harboring either the *RAD30* gene or one of three different polymerase region mutations, *D30A*, *E39A* and *D155A*, known to completely abolish the polymerase function of Polη *in vitro* (Figure 5A), were introduced into *rad30Δ* yeast strains containing the Scc1^NC^ system (Figure 1E). Deletion of *RAD30* renders the cells UV sensitive, which was overcome by expression of the *pRAD30*-plasmid, but not by any of the plasmids with a mutated *RAD30* gene, indicating that they are polymerase dead also *in vivo* (Figure 5B). In Figure 5C we show that the *pRAD30*-plasmid in addition restores the DI-cohesion formation in the *rad30Δ* strain. In strains containing the D30A and E39A mutants, the same was found despite their UV sensitivity. However, the D155A mutation left the cells incapable of forming DI genome-wide cohesion. The possibility that the observed differences in cohesion were due to timely variations in entry into the cell cycle after the G2 arrest was excluded by the fact that the level of Pds1, the protein that keeps separase inactive until anaphase, and thereby prevents chromatid separation, declined simultaneously in all four strains (Figure 5D). Cohesion proficiency could also depend on variable protein levels caused by the mutations. To analyze this, mutated versions of *RAD30* fused to a myc_13_ tag were introduced into the endogenous *RAD30* loci. Whole cell extracts were prepared from equal numbers of wild-type cells without any myc-tagged protein (*RAD30*), cells harboring *RAD30*-*myc_13_* or the various mutated versions of *rad30-myc_13_*, and protein levels were examined by Western blotting. As can be seen in Figure 5E the differences between the differently mutated *rad30* alleles turned out to be rather modest (Figure 5E). The lower levels of the Polη-D155A and -E39A mutant proteins compared with Polη -D30A could most likely not explain why the Polη-D155A mutant is unable to form DI-cohesion, since the Polη-E39A mutant is expressed to a similar level as Polη-D155A but despite this proficient in DI genome-wide cohesion.

### The role of Polη in DI-cohesion is not dependent on interaction with PCNA

It has been suggested that PCNA is important for establishing cohesion during S phase through physical interaction with Eco1 [14], [44]. PCNA is also known to interact with Polη, via Rad6-Rad18-mediated monoubiquitination of the PCNA K164 lysine residue, in response to UV induced DNA damage. This interaction has been proposed to be fundamentally important for the optimal function of Polη in replication past UV-induced CPDs [33], [45], [46]. Interaction between PCNA and Polη has been shown to occur via the ubiquitin-binding motif (UBZ domain) and the (PCNA-binding) PIP box in Polη (Figure 5A) [47], [48]. By introducing point mutations, at the PCNA K164 (*K164R*) and the Polη UBZ (*D570A*) or PIP (*F627A, F628A*) residues, we analyzed the requirement for interaction between PCNA and Polη for establishing DI genome-wide cohesion in three ways, using the Scc1^NC^ system (Figure 1E). As seen in Figure 5F, none of the mutations caused any defect in the formation of DI-cohesion. Thus, unlike the Polη function during replication bypass of UV damage, which is strongly dependent on PCNA, the function during DI genome-wide cohesion seems to be independent of the same.

### DI genome-wide cohesion deficiency caused by lack of Polη is rescued by postreplicative overexpression of *ECO1*

**Figure 6:**
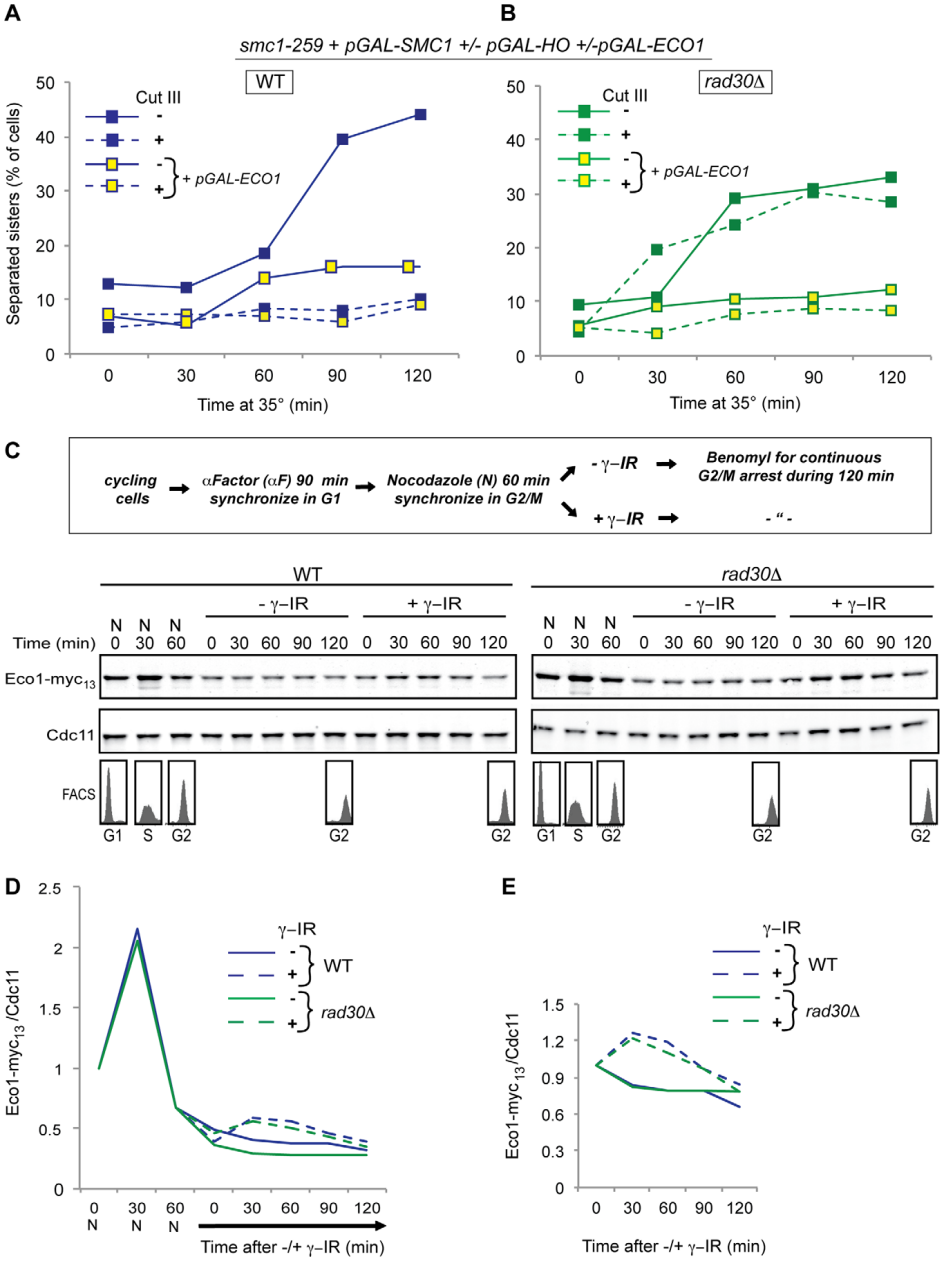
DI-cohesion deficiency caused by lack of Polη is rescued by overexpression of *ECO1*, but Polη is not required for stabilization of Eco1. (A) Formation of DI-cohesion was examined using the experimental system described in Figure 1C. Smc1^WT^ was expressed in all the strains, a plasmid containing *pGAL-ECO1* was introduced into half of the strains and a DSB was induced at the *MAT* locus on Chr. III, in indicated sample series. Samples for detection of sister separation by the TetR-GFP/Tet-O were collected at indicated time points, and separation scored in ≥200 cells per time point. (B) DI-cohesion was determined as in (A) in corresponding strains with *rad30Δ*. (A, B) Blue lines represent WT and green lines *rad30Δ* cells. Dashed lines indicate damage induction. (C) A schematic outline of the experimental set up used for C-E is shown. Eco1-myc_13_ levels were determined by Western blotting on protein extracts prepared at indicated time points from WT and *rad30Δ* cells, in the absence or presence of γ-IR (150 Gy). Cdc11 was used as loading control. FACS profiles from selected time points are presented to show the cell cycle distribution throughout the experiment. One representative experiment (from two performed) is shown. (D) Quantification of the relative Eco1 level, normalized to Cdc11. The time point 0 h nocodazole (0N) for each strain is set to 1. (E) Quantification of the relative Eco1 level, normalized to Cdc11 with the time point 0 h +/− γ-IR set to 1 in each strain.

The possible synergistic functionality of Eco1 and Polη, based on the fact that their orthologs in *S. pombe* are expressed as the fusion protein Eso1 [26], suggested that they are reciprocally dependent on each other for efficient formation of DI genome-wide cohesion. If Polη is supporting the function of Eco1, previously shown to be the limiting factor for DI-cohesion in general [23], excess amounts of Eco1 should rescue the DI genome-wide cohesion defect caused by *rad30Δ*. To test this, we introduced a plasmid containing *ECO1* under control of the *GAL* promoter into strains void of Polη that contain the *smc1^ts^*/Smc1^WT^ system for analyzing DI-cohesion (Figure 1C). With this experimental setup, we not only corroborated the notion that excess amounts of Eco1 can induce cohesion in G2 in the absence of DNA damage (Figure 6A) [23], but also showed that in this situation Polη was no longer required for formation of cohesion, neither in the presence nor in the absence of damage (Figure 6B). Cohesion establishment is normally inactivated after S phase is completed, presumably by the degradation of Eco1. However, in response to DNA damage, this inhibition is overcome by stabilization of Eco1 [49]. We thus tested whether this was the mechanism by which Polη influences the establishment of cohesion in response to DNA damage. WT and *rad30Δ* cells containing myc-tagged Eco1 (Eco1-myc_13_) were arrested in G1 by alpha factor (αF) and synchronously released into a subsequent G2 arrest (Figure 6C). Samples for preparation of protein extracts were withdrawn at indicated time points, and analyzed by Western blotting (Figure 6C). Upon entry into S phase the levels of Eco1 markedly increased (Figure 6C, 6D). These were reduced again at entry into G2 and continued to decline during G2 arrest unless damage was induced. The level of stabilization did not, however, differ between WT and *rad30Δ* strains (Figure 6C, 6D). Thus, the functional importance of Polη for DI genome-wide cohesion is not based on Eco1 stabilization.

### Implications for the action of Polη in Eco1-dependent acetylation of Scc1

**Figure 7:**
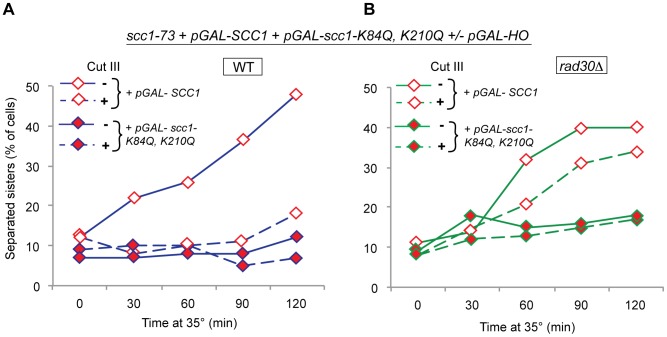
Implications for the action of Polη in Eco1-dependent acetylation of Scc1. (A) Formation of DI- cohesion was examined using the experimental system described in Figure 1C, except that instead of *smc1^ts^* a ts allele of the Scc1 component of cohesin was utilized (*scc1-73*). From introduced plasmids either the *pGAL-SCC1* or an acetyl mimic Scc1 version, *pGAL-scc1-K84Q, K210Q* were expressed. A DSB was induced at the *MAT* locus on Chr. III by HO in indicated sample series. Sister separation was analyzed in samples collected at the indicated time points, by the TetR-GFP/Tet-O-system, and separation scored in ≥200 cells per time point. (B) DI-cohesion was determined as in (A) in corresponding strains with *rad30Δ*. (A, B) Blue lines represent WT and green lines *rad30Δ* cells. Dashed lines indicate damage induction.

Acetylation of the cohesin components, Smc3 during S phase and Scc1 during G2, is potentially the main function of Eco1 during cohesion establishment [10], [11], [12], [25]. We therefore wanted to investigate the Eco1 dependent acetylation events in *rad30Δ* cells. Using an Smc3-acetylation specific antibody [50], [51], we saw, as expected from the finding that Polη seems to be dispensable for S phase cohesion, an upregulation of Smc3 acetylation at the entrance to S phase in both WT and *rad30Δ* cells. The Smc3 acetylation level was in essence maintained during a G2 arrest, both in the absence and in the presence of γ-IR induced DSBs (Figure S4A, S4B, and data not shown). Acetylation of Scc1 in G2, in response to damage has, to our knowledge, not been demonstrated directly [25]. However, an acetyl-mimic version of *SCC1* (*pGAL-scc1-K84Q, K210Q*) has been shown to enable cohesion formation genome-wide during G2 in the absence and presence of DSB induction [25]. Importantly, *pGAL-scc1-K84Q, K210Q* also rescued the DI genome-wide cohesion defect in *rad30Δ* strains, while overexpressed *SCC1 (pGAL-SCC1)* did not (Figure 7A, 7B), indicating that Polη is indeed vital for the acetylation of Scc1 by Eco1.

### DI genome-wide cohesion is important for correct chromosome segregation

**Figure 8:**
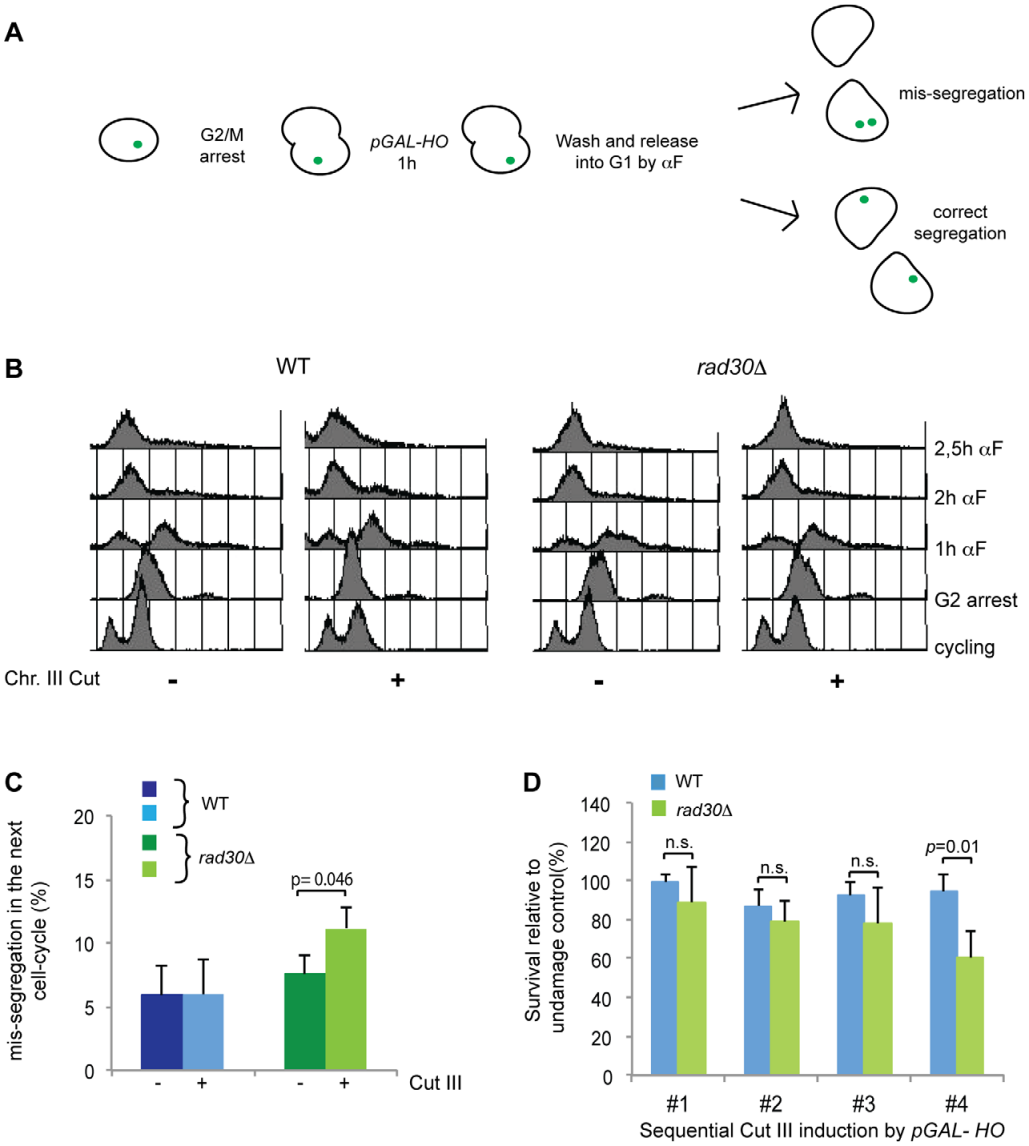
DI-cohesion is required for correct chromosome segregation. (A) Experimental setup of the chromosome segregation assay. Logarithmically growing cells were arrested in G2/M by addition of nocodazole. A DSB was induced at the *MAT* locus on Chr. III by *pGAL-HO*. After 60 min, cells were washed and released from the G2/M arrest into fresh medium containing alpha factor (αF). When 90–100% of the cells had reached G1, samples were scored for chromosome segregation using the TetR-GFP/Tet-O system on Chr. V. (B) FACS profiles showing the G2/M arrest, the subsequent release and the final arrest in G1, in WT and *rad30Δ* strains in the presence or absence of DSB as indicated. (C) The graph shows a comparison of the percent of mis-segregated chromosome V in the absence and presence of DSB on Chr. III, in WT and *rad30Δ* strains as indicated. The error bars show SD values calculated from ≥ three individual experiments. Significant difference is indicated by actual p value (students *t-test*). (D) Survival after repeated rounds of DSB induction by expression of *pGAL-HO* in G2 arrested cells of indicated genotypes.

A possible function for DI genome-wide cohesion, if not important for DSB repair, could be to reinforce the cohesion in undamaged regions of the genome, during an extended G2 arrest as part of a checkpoint response induced by DNA damage, potentially to ensure proper chromosome segregation at anaphase. This issue has been addressed previously, whereby different Eco1 mutated strains that were also defective in S phase cohesion and in DSB repair were analyzed. When a ts allele of *ECO1* (*eco1-1*) that has a very high background of precocious sister separation was used it was concluded that the absence of functional Eco1 during G2 did not cause increased mis-segregation in the subsequent cell cycle. The substantial background level of mis-segregation could however have masked limited differences. In a different *eco1* (eco^ack-^) mutated strain, a threefold increase in loss of unbroken chromosomes after break induction was found [10], [22], [23], [52], [53]. In *rad30Δ* cells there is no deficiency in S phase cohesion and therefore we have the opportunity to specifically measure the effect of absent DI genome-wide cohesion. In WT or *rad30Δ* cells arrested in metaphase by nocodazole a DSB was induced at the *MAT* locus on Chr. III by activation of *pGAL-HO* or not. After one hour the cells were released from the G2/M arrest and subsequently arrested in G1 by addition of αF (Figure 8A). When 90–100% of the cells had reached G1 (Figure 8B), chromosome segregation was determined using the TetR-GFP/Tet-O system on Chr. V. In unchallenged cells, no significant difference between WT and *rad30Δ* cells was apparent. However in response to DSB, *rad30Δ* cells displayed a small but statistically significant increase in chromosomal mis-segregation (Figure 8C). This did not have an immediate negative consequence for survival, but when the mis-segregated population of cells was re-exposed to multiple rounds of induction of a single DSB, we found that after the fourth repetition of damage induction the survival rate of Polη deficient cells compared with WT cells had reduced significantly (Figure 8D). This indeed suggests that the DI genome-wide cohesion has an important function for maintenance of genome integrity and that S phase cohesion is insufficient for correct chromosome segregation in the presence of DNA damage.

## Discussion

It is becoming clear that the cohesin protein complex and its cohesive function are important for genome integrity in multiple ways. Thus, S phase cohesion between sister chromatids is essential both for correct chromosome segregation at anaphase and for postreplicative DSB repair in G2 [3], [19]. It has also been shown that in response to damage, so called DI-cohesion forms in G2 *de novo* that was suggested to be important for repair [21], [22], [23]. In this study we demonstrate that the specialized TLS polymerase, Polη is important specifically for establishment of DI genome-wide cohesion, which is a unique feature of Polη among the translesion synthesis polymerases. The finding that Polη, despite its absolute requirement for formation of DI genome-wide cohesion, was dispensable for postreplicative DSB repair via HR, may be explained by the discovery that Polη is not required for loading of cohesin to the break or for formation of DSB-proximal cohesion. Thus, DI-cohesion is regulated differently in the vicinity of the break compared to genome-wide. Contrary to a recently published study on human cells, where cohesin binding was reinforced after irradiation [43], we noted no significant changes in cohesin binding in WT or *rad30Δ* cells, neither in the absence nor the presence of damage. This either reflects a difference between mammalian and yeast cells, or the fact that we induce a single DSB as opposed to the induction of multiple lesions by γ-IR in the human cells. Regardless, the differential regulation of DSB-proximal cohesion and DI genome-wide cohesion in yeast occurs on another level than cohesin loading.

So, what is the function of Polη during formation of DI genome-wide cohesion? An initial hypothesis was that Polη does for DI-cohesion in G2 what the replicative polymerases do for S phase established cohesion [15]. Polη has been reported to fill in gaps left after bulk replication is finished via its TLS function [54]. This type of DNA synthesis, together with a cohesion machinery reactivated by damage, could possibly establish DI-cohesion. However, two main arguments make this scenario unlikely. Firstly, DI-cohesion is independent of ubiquitination of the PCNA K164 residue, which has been shown to be required for TLS by Polη [33], [45], [46]. Secondly, since two out of three tested Polη polymerase dead mutants are proficient in DI genome-wide cohesion we have no indication that it is the polymerase activity that is required for establishing DI-cohesion. It is however intriguing that the D155A mutation in the active site of Polη is sufficient to disable DI-cohesion formation, despite equal UV sensitivity. One explanation for this could be that the mutations affect protein stability, resulting in different Polη protein levels. However, when determining the resulting levels of myc-tagged Polη in cells harboring the differently mutated *rad30-myc* alleles, they were all expressed at comparable levels. The lower levels of the Polη -D155A and -E39A mutant proteins compared with Polη -D30A could not explain why the Polη -D155A mutant is not able to form DI-cohesion, since the Polη -E39A mutant is expressed to a similar level as Polη -D155A but despite this proficient for DI genome-wide cohesion. Interestingly, the D155 amino acid is responsible for liganding one of the two essential Mg^2+^ ions in the active site of the polymerase, which could be crucial for association with chromatin [55]. It is possible that Polη in this manner enables recruitment of Eco1 to chromatin, which could facilitate acetylation events of importance for DI-cohesion [25]. In line with this we found that an acetyl-mimic version of Scc1 was capable of rescuing the DI genome-wide cohesion defect in *rad30Δ* cells, suggesting that Polη is important for the acetylation of Scc1 by Eco1.

Including Polη, a significant number of proteins have now been shown to be important for DI-cohesion without influencing the ability to repair DSBs in G2 [24]. We showed that DSB-proximal cohesion and DI genome-wide cohesion are regulated differently and that it is presumably the DSB-proximal cohesion that is important for DSB repair. This could explain why the exclusive lack of DI genome-wide cohesion, in the absence of Polη, does not affect HR based postreplicative DSB repair. The specific relevance for DI genome-wide cohesion, in contrast to DSB-proximal cohesion, then had to be redefined. Possibly it is formed in response to a DSB activated checkpoint and is important to prevent precocious sister chromatid separation during a prolonged G2/M arrest. Alternatively it is activated to prevent recombinational repair between homologous chromosomes, a risk caused by the increased movements of both damaged and undamaged chromosomes that occur after DNA damage [56]. In line with this, we showed that absence of DI genome-wide cohesion, results in an increased level of chromosomal mis-segregation at entry of the following cell cycle, indicating that DI genome-wide cohesion is important for correct segregation of chromosomes at anaphase. The consequences of aneuploidy are dramatic since aneuploidy is a hallmark of malignant cells. Indeed, when analyzing the outcome of repeated DSB induction in DI genome-wide cohesion deficient cells it became apparent that this has implications on viability. This parallels the recent finding that not only can reduced genome integrity lead to aneuploidy, but aneuploidy in itself can also cause genome instability [57]. Our results demonstrate a novel function for Polη, but also provide important evidence for differential regulation of DSB-proximal and DI genome-wide cohesion. Furthermore, they suggest a functional interaction between budding yeast Eco1 and Polη as implied by the homologous fission yeast fusion protein between the two.

## Materials and Methods

### Yeast strains and plasmids

All strains used are haploid and of W303 origin (*ade2-1, trp1-1, can1-100, leu2-3, leu112, his3-11, 15, ura3-1, RAD5)*. Genetic modifications and names of the individual strains are listed in Table S1. Deletions of genes were performed by conventional one-step replacement of the open reading frame in question, with kanamycin (*kanMX6*), hygromycin (*hphMX4*) or nourseothricin (*natMX4*) resistance, or the *HIS3* gene [58]. Insertion of the HO cut-site at selected positions in the genome was performed as described [21]. For integration close to the TetR-GFP/Tet-O system on Chr. V, the HO cut-site was amplified using primers with restriction sites for HindIII and BamHI, for subsequent ligation into the pAG32-plasmid (Euroscarf) with the *hphMX4* selection marker. The cut-site sequence was then amplified using primers with homologies 4 kb downstream of the *URA3* locus on Chr. V. Presence of a single copy of the HO cut-site at the desired position was confirmed by PCR. Introduction of plasmids into yeast strains was done using standard yeast transformation protocols. *SMC1-myc_13_* was cloned by PCR and inserted downstream of the *GAL1-10* promoter in the YIPlac128 vector [59], and introduced into the *LEU2* locus of *smc1-259* cells as described [21]. For further details on strains used for DI-cohesion detection see below and Figure 1. To generate strains containing the *rad30*-*F627A, F628A* and *rad30-D570A* mutations we used a two-step PCR-based method [60]. The ORF of *RAD30* was amplified from genomic DNA with HindIII and SalI 5′ flanking primers and cloned into pAG25 plasmid (Euroscarf) digested with above-mentioned enzymes. *RAD30* was then amplified from the vector using a forward gene internal primer that bears the desired mutation. The reverse primer is targeted to the plasmid backbone sequence, amplifying the *natMX4* ORF and bears a 3′ flanking sequence targeted to the *RAD30* 3′ untranslated region. The PCR fragment containing the mutant version of *rad30* linked to *natMX4* was then transformed into appropriate yeast strains. Transformants were selected on YEPD plates containing 10 µg/ml nourseothricin (Jena Bioscience/Sigma). Correct integration was confirmed by PCR and the presence of mutation was verified by sequencing. *POL30* containing the K164R mutation was amplified from genomic DNA from cells carrying the *pol30*-*K164R* allele (a kind gift from H. Ulrich) with BamHI and SacI 5′ flanking primers and cloned into the YIplac211 plasmid. The *YIplac211*-*pol30-K164R:URA3* vector was linearized by ClaI digestion and transformed into appropriate cells. Ura^−^ cells were selected on 5-fluoroorotic acid (5-FOA) plates for selection of *URA3* pop-out clones. Correct integration at the *POL30* gene locus and the removal of the *URA3* gene were confirmed by PCR and the presence of *pol30 -K164R* mutation was screened for by increased UV-sensitivity and confirmed by sequencing. Plasmids (kindly provided by L. Prakash) harboring either the *RAD30* gene in the WT version (*pR30.382:LEU2*) or three different polymerase region mutations, D30A (*pR30.12:LEU2*), E39A (*pR30.127:LEU2*) or D155A (*pR30.138:LEU2*) were transformed into *rad30Δ* yeast strains. Transformants were selected on plates lacking leucine, and were then crossed with yeast strains containing the Scc1^NC^ system (Figure 1E). The experimental strains were tested for UV-sensitivity and the presence or absence of mutation was verified by sequencing of the plasmids rescued from each strain. Myc-tagged variants of *rad30* mutants were generated by PCR amplification of the ORF excluding the stop codon of the *rad30* gene from the above mentioned plasmids; *pR30.126:LEU2, pR30.127:LEU2* and *pR30.138:LEU2* and integrated into the pFA6a-myc_13_-kanMX6 vector. The presence of D30A, E39A or D155A mutations was confirmed by sequencing. The variant *rad30-myc_13_:kanMx6* mutants were then amplified from the vectors using a gene internal forward primer. The reverse primer is targeted to the plasmid backbone sequence, amplifying the *kanMX6* ORF and bears a 3′ flanking sequence targeted to the *RAD30* 3′ untranslated region. Resulting PCR fragments were transformed into appropriate yeast strains. The correct integration of respective *rad30* construct was confirmed by PCR and the presence of mutation was screened for by increased UV-sensitivity and confirmed by sequencing. All PCR amplifications were performed using the proofreading enzyme included in the Long Range dNTP Pack (Roche). The YIplac112 plasmid containing the *pGAL-ECO1* construct (a kind gift from K. Nasmyth) was introduced into strains containing the *smc1^ts^*/Smc1^WT^ system for detection of DI-cohesion (Figure 1C). At the end of the experiments performed on strains containing plasmids expressing *pGAL-ECO1*, or different versions of *RAD30* under its endogenous promoter, plasmids were rescued/purified from the cells using miniprep kit (Qiagen) according to manufacturer's recommendations. Thereafter plasmids were transformed into chemically competent *DH5α* bacteria and purified (Invitrogen quick plasmid miniprep kit). To confirm WT or mutated genotypes the purified plasmids were sequenced. To generate strains harboring either the wild-type or the acetyl-mimic version of Scc1 under the galactose promoter, *pGAL-SCC1-HA_6_* and *pGAL-scc1-K84Q, K210Q-HA_6_* were amplified from pPCM87 and pPCM87-K84Q, K210Q respectively (kind gifts from D. Koshland) and integrated into the YIplac128 plasmid. The YIplac128-*pGAL-SCC1-HA_6_*:*LEU2* and YIplac128-*pGAL-scc1-K84Q, K210Q-HA_6_*:*LEU2* vectors were linearized by EcoRV digestion and transformed into appropriate cells.

### UV sensitivity

Strains harboring the *rad30*-*D30A*, -*E39A*, -*D155A* or *RAD30* in the low-copy number *CEN LEU2* vector YCplac111 were grown to logarithmic phase. Cells were resuspended in YEP-media at identical densities and plated in 10-fold dilutions on YEP plates containing 2% glucose (YEPD). The plates were left untreated or exposed to 35–50 J/m^2^ UV irradiation and incubated at 25–30°C in the dark for 3–4 days.

### Experimental systems for detection of damage-induced cohesion

Three types of experimental systems were used to detect formation of DI-cohesion, one based on the expression of *pGAL-SMC1* in cells where the endogenous *SMC1* allele is temperature sensitive (*smc1-279*), one based on the expression of *pGAL-SCC1* in cells where the endogenous *SCC1* allele is temperature sensitive (*scc1-73*), and one based on expression of an noncleavable version of Scc1, *pGAL*-*scc1^NC^*. DNA damage was then induced either by γ-IR (350 Gy, using a Cs^137^ source with a dose rate of 6 Gy/min), or expression of *pGAL-HO* that creates a DSB at the *MAT* locus on Chr. III or an inserted recognition sequence on Chr. V. Break formation on Chr. III or V, as well as break induction and repair after γ-IR were analyzed by Southern blotting after separation of chromosomes by Pulse Field Gel Electrophoresis, using probes against Chr. III or Chr. XVI as described [21]. During the course of the experiments samples were collected at indicated time points for detection of sister separation at the *URA3* locus on Chr. V by the TetR-GFP/Tet-O system, and separation scored in ≥200 cells/time point. The cell cycle distribution was analyzed by FACS. In all experiments the levels of Smc1^WT^ or Scc1^NC^ were confirmed by *in situ* immuno-fluorescence. For more detailed information of each separate experiment se the main text, figures and figure legends.

### In situ immuno-fluorescence staining and FACS analysis

Pds1-myc_13_, Smc1-myc_13_ or Scc1^NC−^HA_6_ levels were analyzed by *in situ* immuno-staining with anti-myc or anti-HA (Roche) antibodies as previously described [21]. FACS samples were in essence prepared as described [5], and analyzed with a Becton Dickinson FACSCalibur, ensuring 10,000 events per samples.

### Pulse field gel electrophoresis

Pulse field gel electrophoresis was used for verification of induction of a DSB at HO cut-sites on Chr, III, V and VI as well as efficiency of damage induction by γ-IR. Chromosomes were prepared and separated on 1% agarose gel by Pulse Field Gel Electrophoresis as described [19] (Biorad, Chef DRIII). For best separation in the size range of Chr. III and VI the gel was run at 14°C for 24 hr at 6 V/cm with a 35.4–83.55 s switch time and an included angle of 120°. For separation of Chr. V and XVI the gel was run at 10°C with 90 s switch time, otherwise as for Chr. III.

### DNA repair assay

Wild-type and mutant cells were grown in YEPD and arrested in G2/M by benomyl. Arrested cells were exposed to γ-IR (150 Gy). Samples were isolated before and at indicated time points after γ-IR. Cell cycle distribution was analyzed by FACS and efficiency of DNA damage and repair by PFGE and Southern blotting as described [19], [61]. The ratio of the Chr. XVI remaining at specified times were compared with the Chr. XVI signal measured before γ-IR.

### Colony survival assay after exposure to γ-IR

Cells grown in YEPD at 25°C were arrested in G2/M, by addition of benomyl. The cultures were split in two, and one half was exposed to 150 Gy of γ-IR. 300 of both irradiated and control cells were plated on YEPD plates and colonies counted after two days.

### ChIP-on-chip, ChIP sequencing, and qChIP

ChIP-on-chip was performed on FLAG-tagged Scc1 using anti-FLAG (Sigma) in essence as described [42], [62], using *S. cerevisiae* whole-genome tiling 1.0F arrays (Affymetrix). For analysis of G2 expressed cohesin binding, ChIP was performed on HA-tagged *pGAL-SCC1* with anti-HA (Abcam) essentially as described [63], but after 30 min fixation with 1% formaldehyde at room temperature. The samples were then processed either for ChIP sequencing (ChIP seq) or quantitative real time pcr (qRT-PCR). ChIP seq was performed as described [63], and qRT-PCR was performed using SYBR green (Applied Biosystems) according to manufacturer's instructions with primers for the positions indicated in Figure S3B (Chr. V: 520fo 5′-TCGCGTTTCTTACAGTGGCT-3′, 520re 5′-CAGGTCGCCTAATGAAACAG-3′, 529fo 5′-CAATGTCTGGGGAGAGTACT-3′, 529re 5′-CTCCAAACAGATACACCCTC-3′, 534fo 5′-ACAAGCATCATTCATAGCCT-3′, 534re 5′-ATCGTGGCTAGGACATTTTG-3′, 548fo 5′-GAAAATAGCCGCCCAAGGAT-3′, 548re 5′-CTGTGTATATCCCACCAGAC-3′, ChrVIII: 110fo 5′-CCGACCTCTTCTAATCCAAG-3′, 110re 5′-AGAGATGAGGCTCTCAGACA-3′). Samples were then analyzed on ABI Prism 7000 sequence detection system (Applied Biosystems).

### Chromosome segregation assay

Chromosome segregation was analyzed after activation of *pGAL-HO* using the TetR-GFP/Tet-O system. Cells grown in YEPR at 25°C were arrested in G2/M, by addition of nocodazole. Thereafter 2% galactose was added to half of the cultures to induce *pGAL-HO* for one hour. Cells were then washed and released into YEPD containing alpha factor (αF) for G1 arrest. When 90–100% of the cells were in G1, the percentage of arrested cells with a double GFP signal, representing mis-segregation of Chr. V was determined. Cell cycle progression was checked by FACS, break induction by PFGE and Southern blotting using a radioactive probe hybridizing to Chr. III.

### Colony survival assay following multiple rounds of DSB induction

Cells grown in YEPR at 25°C were arrested in G2/M by addition of benomyl. A single DSB was introduced at the *MAT* locus on Chr. III, by expression of *pGAL-HO* for 90 min in half of the cultures. The cells were then washed once in PBS and 300 of both damaged and control cells were plated on YEPD plates and incubated at 25°C. After two days the numbers of colonies were counted for determination of survival. All surviving cells of each population were collected and resuspended at identical densities in YEPR for continued cultivation. This entire experimental procedure was repeated four times in sequence.

### Protein extract preparation and detection by Western blotting

Protein lysates were prepared with lysis buffer (20 mM Tris-HCl pH 8.0, 10 mM MgCl_2_, 1 mM EDTA, 0.3M (NH_4_)_2_SO_4_ and 5% glycerol) supplemented with 1 mM DTT, 1 mM PMSF and 1× Complete EDTA-free protease inhibitor cocktail tablets (Roche) using glass beads vortexing. Proteins were separated by SDS-PAGE using NuPAGE Bis-Tris Gels (Life Technologies) and transferred by Western Blotting using the same system. Antibodies against myc (Roche), cdc11 y-415 (Santa Cruz Biotechnology) and acetylated Smc3 [50], [51] were used. Antibody detection was done using the Odyssey Infrared Imaging System and quantifications were done using Image Studio 2.0 Software (LI-COR Biosciences).

## Supporting Information

Figure S1 Cell population doubling is not delayed in cells lacking Polη. Logarithmically growing WT or *rad30Δ* cells, expressing pGAL-scc1^NC^ or not, as specified, were diluted to OD600 = 0.1 At indicated time points samples were collected for measurement of OD600 as a value of cell density.
(EPS)

Figure S2 Association of Polη to early Autonomously Replicating Sequences on Chr. VI. Logarithmically growing cells were arrested in G1 by addition of αF, cells were then released into S phase for 60 min in the presence of 0.2M Hydroxyurea when FLAG-tagged Polη was ChIPed with anti-FLAG. The sample was prepared and analyzed by ChIP-chip as previously described by Katou et al [62]. Dark blue peaks indicate significant chromosomal binding sites, on the x-axis chromosomal positions of Chr. VI and on the y-axis log2 of signal strength are indicated.
(EPS)

Figure S3 Polη is not required for loading of cohesin during G2. (A) An example of the genome-wide chromosomal association of HA-tagged Scc1 (*pGAL-SCC1^HA^*) expressed in G2 and analyzed by ChIP-sequencing. Shown is a part of Chr. I. The entire genome-wide map is available at the GEO database, with the reference GSE42655, http://www.ncbi.nlm.nih.gov/geo/query/acc.cgi?acc=GSE42655. On top of each panel is the gene organization in the genomic region illustrated. Horizontal blue lines represent open reading frames, vertical red lines indicate replication origins and green CEN positions. In each panel orange peaks indicate significant chromosomal binding sites, on the x-axis chromosomal positions and on the y-axis log_2_ of signal strength are indicated. The different experimental groups tested are: 12_14/10-11 = WT+DSB (LS80), 12A_14/10-11 = WT - DSB (LS81), 12;1_16/10-11 = *rad30Δ*+DSB (LS82), 12;1B_16/10-11 = *rad30Δ* - DSB (LS83). One representative experiment is shown, n = 2. (B) ChIP in combination with real time PCR (qChIP) for quantitative determination of G2 specific HA-tagged Scc1 binding (*pGAL-SCC1^HA^*) in WT and *rad30Δ* strains in the presence and absence of DSB induction at the *MAT* locus on Chr. III, at indicated known cohesin binding sites on Chr. V and Chr. VIII as well as one non binding site on Chr. V (Chr. V 534). Error bars represent SD calculated on three independent experiments.
(EPS)

Figure S4 Smc3 is acetylated to a similar extent in WT and *rad30Δ* cells. (A) Western blot detection of acetylated Smc3 from G1 to G2 and at indicated time points during a G2 arrest after IR. Detection of the constitutively expressed Cdc11 protein was used as loading control. The cell cycle distribution of the cell population is shown below the WB panels at the different time points. (B) Quantification of A.
(EPS)

Table S1 Genetic modifications of the yeast strains used in this study. All strains used were originally haploid and of W303 origin (*ade2-1, trp1-1, can1-100, leu2-3, 112, his3-11, 15, ura3-1, RAD5, GAL, psi+)*.
(PDF)
