# Supplementary material for: Importance of Polη for Damage-Induced Cohesion Reveals Differential Regulation of Cohesion Establishment at the Break Site and Genome-Wide
Source: PLoS Genet. 2013 Jan 10;9(1):e1003158. doi: 10.1371/journal.pgen.1003158 (PMC3542068; doi:10.1371/journal.pgen.1003158)
Supplement: Table S1 — Genetic modifications of the yeast strains used in this study. All strains used were originally haploid and of W303 origin (ade2-1, trp1-1, can1-100, leu2-3, 112, his3-11, 15, ura3-1, RAD5, GAL, psi+). (PDF) [file pgen.1003158.s005.pdf]

Supplementary material

Importance of Polη for damage-induced cohesion reveals differential regulation of cohesion establishment at the break site and genome-wide

Elin Enervald, Emma Lindgren, Yuki Katou, Katsuhiko Shirahige and Lena Ström

Table S1. Strains used in this study

All strains used are haploid and of W303 origin (*ade2-1, trp1-1, can1-100, leu2-3, 112, his3-11, 15, ura3-1, RAD5, GAL, psi+)*

Strain	Genotype
LS6	*MATa, ECO1-myc$_{13}$:kanMX6*
LS7	*MATa, RAD30-myc$_{13}$:kanMX6*
LS11	*MATalpha, pol30-K167R, ade3::pGAL:HO, leu2::pGAL-scc1-R180D, R268D-HA$_{3}$:LEU2, his3::TetR-GFP:HIS3, ura3::TetOs:URA3*
LS28	*MATa, rad30::kanMX6, rad52::HISMX6, his3::TetR-GFP:HIS3, ura3::TetOs:URA3*
LS29	*MATa, rad30::kanMX6, lig4::HISMX6, his3::TetR-GFP:HIS3, ura3::TetOs:URA3*
LS38	*MATalpha::natMX4, ade3::pGAL:HO, smc1-259, leu2::pGAL-SMC1-myc$_{13}$:LEU2, his3::TetR-GFP:HIS3, ura3::TetOs:URA3*
LS39	*MATalpha::natMX4, ade3::pGAL:HO, 3'URA3::HOcs::hphMX4, smc1-259, leu2::pGAL-SMC1-myc$_{13}$:LEU2, his3::TetR-GFP:HIS3, ura3::TetOs:URA3*
LS40	*MATalpha::natMX4, rad30::kanMX6, ade3::pGAL:HO, smc1-259, leu2::pGAL-SMC1-myc$_{13}$:LEU2, his3::TetR-GFP:HIS3, ura3::TetOs:URA3*
LS41	*MATalpha::natMX4, rad30::kanMX6, ade3::pGAL:HO, 3'URA3::HOcs::hphMX4, smc1-259, leu2::pGAL-SMC1-myc$_{13}$:LEU2, his3::TetR-GFP:HIS3, ura3::TetOs:URA3*
LS61	*MATalpha, rad30-F627A,F628A::natMX4, ade3::pGAL:HO, leu2::pGAL-scc1-R180D, R268D-HA3:LEU2, his3::TetR-GFP:HIS3, ura3::TetOs:URA3*
LS69	*MATalpha::natMX4, ade3::pGAL:HO, 3'URA3::Hocs::hphMX4, smc1-259, his3::TetR-GFP:HIS3, ura3::TetOs:URA3*
LS72	*MATalpha::natMX4, rad30::kanMX6, ade3::pGAL:HO, 3'URA3::HOcs::hphMX4, smc1-259, his3::TetR-GFP:HIS3, ura3::TetOs:URA3,*
LS73	*MATa, rad30::kanMX6, ade3::pGAL:HO, 3'URA3::HOcs::hphMX4, smc1-259*
LS79	*MATa, rad30::kanMX6, ade3::pGAL:HO, his3::TetR-GFP:HIS3, ura3::TetOs:URA3*
LS80	*MATalpha, ade3::pGAL:HO, ura3::pGAL-SCC1-HA$_{6}$:URA3*
LS81	*MATalpha::natMX4, ade3::pGAL:HO, ura3::pGAL-SCC1-HA$_{6}$:URA3*
LS82	*MATalpha, rad30::kanMX6, ade3::pGAL:HO, ura3::pGAL-SCC1-HA$_{6}$:URA3*
LS83	*MATalpha::natMX4, rad30::kanMX6, ade3::pGAL:HO, ura3::pGAL-SCC1-HA$_{6}$:URA3*
LS87	*MATalpha, rev3::hphMX4, ade3::pGAL:HO, leu2::pGAL-scc1-R180D, R268D-HA$_{3}$:LEU2, his3::TetR-GFP:HIS3, ura3::TetOs:URA3*
LS89	*MATalpha, rev1::hphMX4, ade3::pGAL:HO, leu2::pGAL-scc1-R180D, R268D-HA$_{3}$:LEU2, his3::TetR-GFP:HIS3, ura3::TetOs:URA3*
LS105	*MATa, rev1::natMX4, rev3::hphMX4, rad30::kanMX6, his3::TetR-GFP:HIS3, ura3::TetOs:URA3*

LS112	*MATalpha, smc1-259, leu2::pGAL-SMC1-myc$_{13}$:LEU2, trp1::pGAL-ECO1:TRP1, his3::TetR-GFP:HIS3, ura3::TetOs:URA3*
LS114	*MATalpha, ade3::pGAL:HO, smc1-259, leu2::pGAL-SMC1-myc$_{13}$:LEU2, trp1::pGAL-ECO1:TRP1, his3::TetR-GFP:HIS3, ura3::TetOs:URA3*
LS116	*MATalpha, rad30::kanMX6, ade3::pGAL:HO, smc1-259, leu2::pGAL-SMC1-myc$_{13}$:LEU2, trp1::pGAL-ECO1:TRP1, his3::TetR-GFP:HIS3, ura3::TetOs:URA3*
LS118	*MATalpha, rad30::kanMX6, smc1-259, leu2::pGAL-SMC1-myc$_{13}$:LEU2, trp1::pGAL-ECO1:TRP1, his3::TetR-GFP:HIS3, ura3::TetOs:URA3*
LS166	*MATalpha, rad30-D570A:natMX4, ade3::pGAL:HO, leu2::pGAL-scc1-R180D, R268D-HA$_3$:LEU2, his3::TetR-GFP:HIS3, ura3::TetOs:URA3, PDS1-myc$_{18}$:TRP1*
LS173	*MATa, leu2::pGAL:SCC1-HA$_6$:LEU2, scc1-73, his3::TetR-GFP:HIS3, ura3::TetOs:URA3, ade3::pGAL:HO*
LS174	*MATa, leu2::pGAL:scc1-K84Q,K210Q-HA$_6$:LEU2, scc1-73, his3::TetR-GFP:HIS3, ura3::TetOs:URA3, ade3::pGAL:HO*
LS175	*MATa, rad30::kanMX6, leu2::pGAL:SCC1-HA$_6$:LEU2, scc1-73, his3::TetR-GFP:HIS3, ura3::TetOs:URA3, ade3::pGAL:HO*
LS176	*MATa, rad30::kanMX6, leu2::pGAL:scc1-K84Q,K210Q-HA$_6$:LEU2, scc1-73, his3::TetR-GFP:HIS3, ura3::TetOs:URA3, ade3::pGAL:HO*
LS194	*MATalpha::natMX4, rad30::kanMX6, ade3::pGAL:HO, leu2::pGAL-scc1-R180D, R268D-HA$_3$:LEU2, PDS1-myc$_{18}$:TRP1, his3::TetR-GFP:HIS3, ura3::TetOs:URA3*
LS234	*MATa, rad30-D30A-myc$_{13}$:kanMX6*
LS236	*MATa, rad30-E39A-myc$_{13}$:kanMX6*
LS237	*MATa, rad30-D155A-myc$_{13}$:kanMX6*
LS240	*MATa, ECO1-myc$_{13}$:kanMX6, rad30::natMX4*
CB67	*MATa*
CB328	*MATalpha, rad52::HIS3MX6*
CB423	*MATa, leu2:: pGAL-scc1-R180D, R268D-HA$_3$:LEU2, his3::TetR-GFP:HIS3, ura3::TetOs:URA3*
CB469	*MATalpha, smc1-259, leu2::pGAL-SMC1-myc$_{13}$:LEU2, his3::TetR-GFP:HIS3, ura3::TetOs:URA3*
CB479	*MATalpha, ade3::pGAL:HO, smc1-259, leu2::pGAL-SMC1-myc$_{13}$:LEU2, his3::TetR-GFP:HIS3, ura3::TetOs:URA3*
CB480	*MATa, ade3::pGAL:HO, smc1-259, leu2::pGAL-SMC1-myc$_{13}$:LEU2, his3::TetR-GFP:HIS3, ura3::TetOs:URA3*
CB506	*MATalpha, ade3::pGAL:HO, leu2::pGAL-scc1-R180D, R268D-HA$_3$:LEU2, PDS1-myc$_{18}$:TRP1, his3::TetR-GFP:HIS3, ura3::TetOs:URA3*
CB507	*MATa, ade3::pGAL:HO, leu2::pGAL-scc1-R180D, R268D-HA$_3$:LEU2, PDS1-myc$_{18}$:TRP1, his3::TetR-GFP:HIS3, ura3::TetOs:URA3*
CB524	*MATa, ade3::pGAL:HO, PDS1-myc$_{18}$:TRP1, his3::TetR-GFP:HIS3, ura3::TetOs:URA3*
CB582	*MATa, ade3::pGAL:HO, smc1-259, his3::TetR-GFP:HIS3, ura3::TetOs:URA3*
CB586	*MATalpha::kanMX6, ade3::pGAL:HO, leu2::pGAL-scc1-R180D, R268D-HA$_3$:LEU2, PDS1-myc$_{18}$:TRP1, his3::TetR-GFP:HIS3, ura3::TetOs:URA3*

CB612	*MATa, rad30::kanMX6, his3::TetR-GFP:HIS3, ura3::TetOs:URA3*
CB625	*MATalpha, rad30::kanMX6, ade3::pGAL:HO, leu2::pGAL-scc1-R180D, R268D-HA$_3$:LEU2, PDS1-myc$_{18}$:TRP1, his3::TetR-GFP:HIS3, ura3::TetOs:URA3*
CB626	*MATalpha, rad30::kanMX6, ade3::pGAL:HO, smc1-259, leu2::pGAL-SMC1-myc$_{13}$:LEU2, his3::TetR-GFP:HIS3, ura3::TetOs:URA3*
CB627	*MATa, rad30::kanMX6, ade3::pGAL:HO, smc1-259, leu2::pGAL-SMC1-myc$_{13}$:LEU2, his3::TetR-GFP:HIS3, ura3::TetOs:URA3*
CB697	*MATalpha, rad30::kanMX6 smc1-259, leu2::pGAL-SMC1-myc$_{13}$:LEU2, his3::TetR-GFP:HIS3, ura3::TetOs:URA3*
CB698	*MATa, rad30::kanMX6 smc1-259, leu2::pGAL-SMC1-myc$_{13}$:LEU2, his3::TetR-GFP:HIS3, ura3::TetOs:URA3*
CB1206	*MATa, rad30::kanMX6, pR30.126:LEU2, ade3::pGAL:HO leu2::pGAL-scc1-R180D, R268D-HA$_3$:LEU2, PDS1-myc$_{18}$:TRP1, his3::TetR-GFP:HIS3, ura3::TetOs:URA3*
CB1210	*MATa, rad30::kanMX6, pR30.127:LEU2, ade3::pGAL:HO, leu2::pGAL-scc1-R180D, R268D-HA$_3$:LEU2, PDS1-myc$_{18}$:TRP1, his3::TetR-GFP:HIS3, ura3::TetOs:URA3*
CB1214	*MATa, rad30::kanMX6, pR30.138:LEU2, ade3::pGAL:HO, leu2::pGAL-scc1-R180D, R268D-HA$_3$:LEU2, PDS1-myc$_{18}$:TRP1, his3::TetR-GFP:HIS3, ura3::TetOs:URA3*
CB1218	*MATa, rad30::kanMX6, pR30.382:LEU2, ade3::pGAL:HO leu2::pGAL-scc1-R180D, R268D-HA$_3$:LEU2, PDS1-myc$_{18}$:TRP1, his3::TetR-GFP:HIS3, ura3::TetOs:URA3*
CB1279	*MATa, lig4::HIS3MX6*
